# Epithelial-Immune Cell Crosstalk Determines the Activation of Immune Cells In Vitro by the Human Cathelicidin LL-37 at Low Physiological Concentrations

**DOI:** 10.3390/biom13091316

**Published:** 2023-08-28

**Authors:** Ivan V. Bogdanov, Maria A. Streltsova, Elena I. Kovalenko, Alexander M. Sapozhnikov, Pavel V. Panteleev, Tatiana V. Ovchinnikova

**Affiliations:** 1 M.M. Shemyakin & Yu. A. Ovchinnikov Institute of Bioorganic Chemistry, Russian Academy of Sciences, 117997 Moscow, Russia; mstreltsova@mail.ru (M.A.S.); kovalenelen@gmail.com (E.I.K.); amsap@mail.ru (A.M.S.); p.v.panteleev@gmail.com (P.V.P.); ovch@ibch.ru (T.V.O.); 2 Department of Biotechnology, I.M. Sechenov First Moscow State Medical University, 119991 Moscow, Russia

**Keywords:** LL-37, human cathelicidin, host defense peptide, immunomodulatory action, cytokines, Caco-2 cells, epithelial immunity, xMAP

## Abstract

The only human cathelicidin, LL-37, is a host defense antimicrobial peptide with antimicrobial activities against protozoans, fungi, Gram(+) and Gram(−) bacteria, and enveloped viruses. It has been shown in experiments in vitro that LL-37 is able to induce the production of various inflammatory and anti-inflammatory cytokines and chemokines by different human cell types. However, it remains an open question whether such cytokine induction is physiologically relevant, as LL-37 exhibited its immunomodulatory properties at concentrations that are much higher (>20 μg/mL) than those observed in non-inflamed tissues (1–5 μg/mL). In the current study, we assessed the permeability of LL-37 across the Caco-2 polarized monolayer and showed that this peptide could pass through the Caco-2 monolayer with low efficiency, which predetermined its low absorption in the gut. We showed that LL-37 at low physiological concentrations (<5 μg/mL) was not able to directly activate monocytes. However, in the presence of polarized epithelial monolayers, LL-37 is able to activate monocytes through the MAPK/ERK signaling pathway and induce the production of cytokines, as assessed by a multiplex assay at the protein level. We have demonstrated that LL-37 is able to fulfill its immunomodulatory action in vivo in non-inflamed tissues at low physiological concentrations. In the present work, we revealed a key role of epithelial-immune cell crosstalk in the implementation of immunomodulatory functions of the human cathelicidin LL-37, which might shed light on its physiological action in vivo.

## 1. Introduction

Human and animal cathelicidins comprise one of the largest and most well-studied classes of antimicrobial host defense peptides (HDPs) in higher vertebrates [1]. Cathelicidins represent not only the front line of host defense against infection but are also involved in innate and acquired immunity [2]. Cathelicidin genes have been studied in many mammalian species, including humans, cows, horses, pigs, sheep, goats, rabbits, mice, guinea pigs, and others [3]. A number of species have multiple cathelicidin genes encoding different peptides [4,5], but humans and mice contain a single functional cathelicidin gene in each genome. The only human cathelicidin LL-37 sequence begins with two leucine amino acid residues and is composed of 37 a.a. residues, giving the abbreviation LL-37 to this peptide. It is found in secretory granules of neutrophils in its 18-kDa inactive prepropeptide form designated as hCAP-18, which includes the C-terminal cationic antimicrobial peptide cathelicidin LL-37 and the neutralizing N-terminal pro-domain, consisting of the signal peptide (30 a.a.) and the highly conserved pro-sequence (103 a.a.) called the cathelin-like domain [4,6]. Degranulation and release of hCAP-18 occur via stimulation by pathogen-associated molecular patterns (PAMPs), such as lipopolysaccharide (LPS). After the peptide release, endoproteolytic cleavage of the C-terminal cathelicidin LL-37 activates the peptide. In addition to constitutive expression in neutrophils, hCAP-18 is also secreted by different cell types, such as keratinocytes, epithelial cells, natural killer (NK) cells, monocytes/macrophages, dendritic cells, mast cells, lymphocytes, mesenchymal stem cells, and bone marrow stroma [7]. LL-37 is believed to play an important role in epithelial immunity under pathogen infection, especially in airway epithelium and intestinal mucosal surfaces [8,9]. For instance, LL-37/hCAP-18 was shown to be expressed in epithelial cells of the upper crypts of the normal human colon in vivo [10]. LL-37 is able to increase the expression of epithelial tight junction proteins (claudin-1, occluding, ZO-1, and ZO-2), which are known to be associated with intestinal barrier function [10].

LL-37 demonstrates a broad spectrum of antimicrobial activities against Gram(+) and Gram(−) bacteria, including *S. aureus*, *M. tuberculosis*, and *L. monocytogenes*; viruses such as herpes simplex virus type 1 (HSV-1) and adenovirus; fungi such as *C. albicans*; and some parasitic protozoans such as *Leishmania* sp. [11]. Apart from a broad spectrum of antimicrobial activities, LL-37 has potent immunomodulatory properties. For instance, LL-37 was shown to be involved in the induction of both pro- and anti-inflammatory responses in the immune system. Pro-inflammatory responses are usually linked with the stimulation of chemotaxis (via recruiting and activating neutrophils), immune cell differentiation, and the induction of adaptive immunity responses, whereas anti-inflammatory responses are implemented through the inhibition of cytokine release and direct scavenging of bacterial endotoxins [7,12]. It has been shown that in in vitro experiments, the human cathelicidin, LL-37, was able to induce the production of various cytokines and chemokines by different human cell types. For instance, LL-37 has been shown to induce the production of the inflammatory cytokines IL-1β and TNFα by macrophages and monocytes, the inflammatory IL-36 by keratinocytes, upregulate anti-inflammatory receptors such as TGF-βR, and stimulate the secretion of the anti-inflammatory cytokine IL-10 by macrophages [13]. However, it remains an open question whether such induction of cytokine production occurs in vivo, or, in other words, if this immunomodulatory action of LL-37 is biologically relevant, as most of the experiments were conducted using LL-37 at concentrations that were much higher (>20 μg/mL), than those of LL-37 in non-inflamed tissues (1–5 μg/mL).

LL-37 has been studied in a number of clinical trials on humans [14,15]. It has been anticipated that research interest in the therapeutic potential of LL-37 will continue to expand, and there might be new discoveries in the near future [16]. However, there is still a lack of data regarding the absorption of LL-37 in the gut in vivo or its permeability through the gastrointestinal barrier in vitro.

The aim of this study is to assess the in vitro permeability potential of human cathelicidin LL-37 across the Caco-2 polarized monolayer to roughly estimate its absorption properties in the human intestine and also to study the immunomodulatory properties of LL-37 at low physiological concentrations (up to 5 μg/mL) on different human cell cultures in the presence of an epithelial barrier in order to evaluate a possible role of LL-37 in epithelial immunity.

## 2. Materials and Methods

### 2.1. Materials

LL-37 (M.w. 4493) (of >98% purity) was produced using standard solid-phase peptide synthesis. This peptide was kindly provided by Dr. Maxim N. Zhmak and subsequently purified by high-performance liquid chromatography (RP-HPLC) on a semi-preparative reversed-phase column, ReproSil-Pur C18-AQ, 5-μm particle size, pore size 120 Å, 10 × 250 mm (Dr. Maisch GmbH, Ammerbuch, Germany). RP-HPLC was performed at a flow rate of 2 mL/min in a linear gradient from 5 to 80% (*v*/*v*) of acetonitrile in water with the addition of 0.1% trifluoroacetic acid (TFA) for 60 min.

### 2.2. Human Cell Lines and Cultures

#### 2.2.1. Growing of Caco-2 Epithelial Barriers

Caco-2 cell line (ATCC HTB-37) was cultured in a humidified CO_2_-incubator (5% CO_2_, 37 °C) in the complete DMEM/F12 (1:1) medium (Gibco, Waltham, MA, USA) containing 10% fetal bovine serum (FBS, Gibco) and 1X antibiotic–antimycotic solution (Gibco). To stabilize the cell phenotype, Caco-2 cells have been subcultivated three times before seeding on the filter supports. Then, Caco-2 cells were seeded at a density of 2 × 10^5^ cells/cm^2^ onto 24-well Millicell cell culture inserts (PC, 0.4-μm pore size, 0.6 cm^2^ surface area, cat. #PIHP01250) (Millipore, Burlington, MA, USA), precoated with 0.2% bovine gelatin (Sigma-Aldrich, Saint Louis, MO, USA). After seeding, Caco-2 cells were grown in the same complete medium with re-feeding every 2–3 days until polarization of the monolayer occurred. The functional polarity of the monolayers was developed when transepithelial electrical resistance (TEER) between the apical and basolateral surfaces of the monolayers reached 400 Ω/cm^2^ as measured by the electrical resistance system Millicell-ERS Voltohmmeter (Millipore, Burlington, MA, USA). Grown cell monolayers were used in transport and cytokine production experiments between the 21st and 29th days.

#### 2.2.2. Generation of Mature Monocyte-Derived Dendritic Cells (moDCs)

Peripheral blood mononuclear cells (PBMCs) from a healthy donor were purchased from the American Type Culture Collection (ATCC PCS-800-011). Blood monocytes were isolated from PBMCs by plastic adhesion [17]. Mature monocyte-derived dendritic cells (moDCs) were generated in vitro from peripheral blood monocytes, according to Nair et al. [18]. Briefly, monocytes were cultured in a humidified CO_2_-incubator (5% CO_2_, 37 °C) in the complete RPMI-1640 medium (Gibco) with 10% FBS, antibiotics, and supplemented with a cytokine cocktail containing 500 U/mL rhIL-4 and 800 U/mL rhGM-CSF (both Sci-Store, Moscow, Russia). On the third day, the medium was replaced by the same fresh medium with FBS, antibiotics, and the cytokine cocktail. Induction of maturation of the immature monocyte-derived dendritic cells was performed on the 5th day by replacing the medium with the complete RPMI-1640 medium (Gibco) with 10% FBS and antibiotics, supplemented with a cytokine cocktail containing 500 U/mL rhIL-4, 800 U/mL rhGM-CSF (both Sci-Store, Moscow, Russia), and 100 U/mL rhTNF-α (Sigma-Aldrich), and subjected to subsequent incubation of the culture for 4 more days. 

#### 2.2.3. Isolation of NKT Cells

NKT cells (CD3^+^CD56^+^) were isolated from PBMCs using the FACSVantageDiVa cell sorter (Becton Dickinson, Franklin Lakes, NJ, USA), equipped with 405, 488, and 643 nm lasers (Supporting Materials, Figure S1). PBMCs were re-suspended in the FACS buffer (PBS containing 0.5% BSA and 2 mM EDTA) and labeled with monoclonal mouse anti-human CD3-APC (clone UCHT1, Beckman Coulter, Pasadena, CA, USA) and mouse anti-human CD56-PE/Cy7 (clone N901, Beckman Coulter) antibodies to isolate NKT cells (CD3^+^CD56^+^). 

Sorted NKT cells were expanded by stimulation with Dynabead Human T-Expander CD3/CD28 magnetic beads (Gibco) in RPMI-1640 medium containing 10% FBS, 1X antibiotic–antimycotic solution, 100 U/mL IL-2 (Gibco), and 50 ng/mL IL-7 (Sci-Store). CD3/CD28 magnetic beads were subsequently removed with a magnet. Expanded NKT cells were washed out of the cytokines and cryopreserved.

### 2.3. Labeling of LL-37 with Fluorescein Isothiocyanate (FITC)

LL-37 was labeled with fluorescein isothiocyanate isomer I (FITC) (Sigma-Aldrich). For coupling with FITC, 0.5 mg of LL-37 in 50 µL of DMSO was first mixed with 200 µL of the carbonate–bicarbonate coupling buffer (0.1 M sodium carbonate, 0.1 M sodium bicarbonate, pH 10.4). Then, 4 mg of FITC in 200 µL of DMSO was added to the solution, and the resultant mixture was incubated in the dark at room temperature (20–22 °C) for 2 h. FITC-LL-37 was purified by using a PD10 gel-filtration column (GE Healthcare, Chicago, IL, USA) pre-equilibrated with PBS.

### 2.4. Transport of FITC-LL-37 through the Caco-2 Epithelial Monolayer

The penetration capability of FITC-LL-37 was investigated using Caco-2 cells as an in vitro model of the intestinal epithelial barrier, grown on permeable Millicell cell culture inserts. Transport of the compound from both sides of the monolayer was determined in the transport buffer (Hank’s balanced salt solution, containing 25 mM HEPES, 1 mM MgCl_2_, 1 mM CaCl_2_, and 10 mM D(+)glucose, pH 7.4). To study the transport in the “apical-to-basolateral” (A → B) direction, 0.7 mL of the transport buffer (pH 7.4) was added to the wells of a 24-well plate, and 5 µM compound in the same transfer buffer was added to the apical chamber. At the same time, to study the transport in the “basolateral-to-apical” (B → A) direction, 0.4 mL of the transport buffer was added to the apical chamber, while 5 µM compound in 0.7 mL of the same transfer buffer was added to the wells of the 24-well plate from the basolateral (serosal) side of the monolayer. The transfer buffer and the donor solution (5 µM of LL-37 in the transfer buffer) were pre-heated to 37 °C immediately prior to the experiment. Transport of LL-37 through Caco-2 polarized monolayers was conducted for 90 min in 4 independent inserts for each direction.

Transfer of LL-37 through Caco-2 monolayers under steady-state conditions was evaluated mathematically for each direction and each insert by the apparent permeability coefficient (P_app_), calculated according to the following Equation (1):

P_app_ = ΔC/Δt × (V/(A × C_i_)) (1)

where ΔC/Δt is the solute flux (nM/s) across the barrier, V is the volume of the acceptor chamber, A is the effective filter area of the well (cm^2^), and C_i_ is the initial concentration of the donor solution (nM). Analysis of the bidirectional “apical-to-basolateral” (A → B) and “basolateral-to-apical” (B → A) transport also included calculation of uptake (UR) and efflux (ER) ratios according to the following Equations (2) and (3):

UR = P_app_(A → B)/P_app_(B → A) (2)

ER = P_app_(B → A)/P_app_(A → B) (3)

The integrity of the monolayers was checked by measuring TEER at the beginning and after the end of the transport experiment.

### 2.5. Stimulation of Human Cell Cultures with LL-37

Primary blood monocytes, moDCs, and NKT cells were seeded in 600 µL of complete RPMI-1640 with 10% Human AB serum (Capricorn Scientific, Ebsdorfergrund, Germany) and supplemented with 1X antibiotic–antimycotic solution into the wells of 24-well plates at a density of 3–7 × 10^5^ cells/well 24 h before the experiment. Then, 24 h later, the medium in each well was replaced by the same medium with 1 µM of LL-37 for the sample wells or fresh medium alone for the control wells, and the permeable inserts with the Caco-2 monolayer with TEER > 400 Ω × cm^2^, containing 400 μL of the complete medium in their apical chamber, were immediately placed into the appropriate wells. Also, inserts with the Caco-2 monolayer were placed in cell-free wells of 24-well plates in the same complete RPMI-1640 medium with or without 1 µM of LL-37 as controls. Cells were kept in a CO_2_-incubator (5% CO_2_, 37 °C) for another 24 h. Then, culture medium from each well was collected, centrifuged, and frozen at −70 °C.

### 2.6. Multiplex Assay to Quantitatively Evaluate Cytokines, Chemokines, and Growth Factor Levels in Culture Supernatants

To quantitatively evaluate a broad spectrum of analytes secreted by human cell cultures at the protein level, multiplex xMAP technology was used. The chosen panel (cat. #HCYTA-60K-PX48, Merck, Darmstadt, Germany) included 48 different magnetic beads with primary antibodies to the following cytokines, chemokines, and growth factors: CCL11 (Eotaxin-1), Flt-3 ligand, CX3CL1 (Fractalkine), sCD40L, FGF-2, EGF, GROα, GM-CSF, G-CSF, IL-1RA, IL-1α, IL-1β, IL-2, IL-3, IL-4, IL-5, IL-6, IL-7, CXCL8 (IL-8), IL-9, IL-10, IL-12p40, IL-12p70, IL-13, IL-15, IL-17A (CTLA8), IL-17E (IL-25), IL-17F, IL-18, IL-22, IL-27, IFNγ, IFNα2, CXCL10 (IP-10), CCL2 (MCP-1), CCL7 (MCP-3), CCL22 (MDC), CXCL9 (MIG), CCL3 (MIP-1α), CCL4 (MIP-1β), CCL5 (RANTES), M-CSF, PDGF-AA, PDGF-AB/BB, TNFα, TNFβ, TGFα, and VEGF-A. The assay was performed in accordance with the manufacturer’s instructions with overnight incubation of the samples with primary antibodies, and the plate was read on a MAGPIX System (Merck) using the xPONENT 4.2 software (Merck). The acquired fluorescence data were analyzed by the MILLIPLEX Analyst v5.1 software (Merck), which realized an advanced curve-fitting algorithm. Levels of the analytes in control and experimental samples were compared with an unpaired two-sample *t*-test using GraphPad Prism v.8.0.1 (GraphPad Software, Inc., San Diego, CA, USA). The *p*-values ≤ 0.05 were considered significant.

### 2.7. Analysis of Cell Signaling Pathways

Primary blood monocytes were isolated from PBMCs by plastic adhesion in the wells of a 24-well plate 24 h prior to the experiment. For that, 2 × 10^6^ PBMC were seeded in each well in 2 mL of RPMI-1640 without supplements and kept for 1 h in a humidified CO_2_-incubator (5% CO_2_, 37 °C). Then, the adhered monocytes were thoroughly washed 3 times with the culture medium without supplements, and 600 µL of the complete RPMI-1640, supplemented with 10% Human AB serum, was added into each well. Then, 24 h later, the medium in the wells of a 24-well plate was replaced by a fresh medium with or without 1 µM of LL-37, and the permeable inserts with Caco-2 monolayers with TEER > 400 Ω × cm^2^, containing 400 μL of the complete medium in their apical chamber, were immediately placed into the appropriate wells for 2 h. Then, 2 h later, the culture medium and the inserts were carefully removed, and the cells in each well were washed with PBS and lysed in 100 µL of MILLIPLEX MAP Lysis Buffer (cat. #43-040, Merck, Darmstadt, Germany), containing phosphatase inhibitors including 1 mM sodium orthovanadate (Na_3_VO_4_). To prevent protein degradation, a protease inhibitor cocktail (cat. #535140, Merck) was freshly added to the MILLIPLEX MAP Lysis Buffer. Cell lysate preparation was performed in accordance with the manufacturer’s instructions.

The phosphorylation of the following key cell signaling kinases and transcription factors was measured in cell lysates by using the MILLIPLEX multi-pathway magnetic bead 9-plex kit (#48-680MAG, Merck), containing phosphorylation state-specific antibodies: ERK/MAP kinase 1/2 (Thr185/Tyr187), Akt (Ser473), STAT3 (Ser727), JNK (Thr183/Tyr185), p70S6 kinase (Thr412), NF-κB (Ser536), STAT5A/B (Tyr694/699), CREB (Ser133), and p38 (Thr180/Tyr182). β-Tubulin MAPmate magnetic beads (#46-713MAG, Merck) were used as an assay normalization control to normalize the total protein level between the samples. A multiplex assay was carried out in accordance with the manufacturer’s instructions with overnight incubation of the samples with primary antibodies to phosphorylated signaling proteins on the MAGPIX system (Merck) using the xPONENT 4.2 software (Merck). The final analysis of the acquired fluorescence data was performed with the MILLIPLEX Analyst v5.1 software (Merck). Normalized MFI values were compared with an unpaired two-sample *t*-test using GraphPad Prism v.8.0.1 (GraphPad Software). The *p*-values ≤ 0.05 were considered significant.

## 3. Results

### 3.1. The Human Host Defense Peptide LL-37 Is Able to Pass across the Intestinal-Like Caco-2 Epithelial Barrier

**Figure 1:**
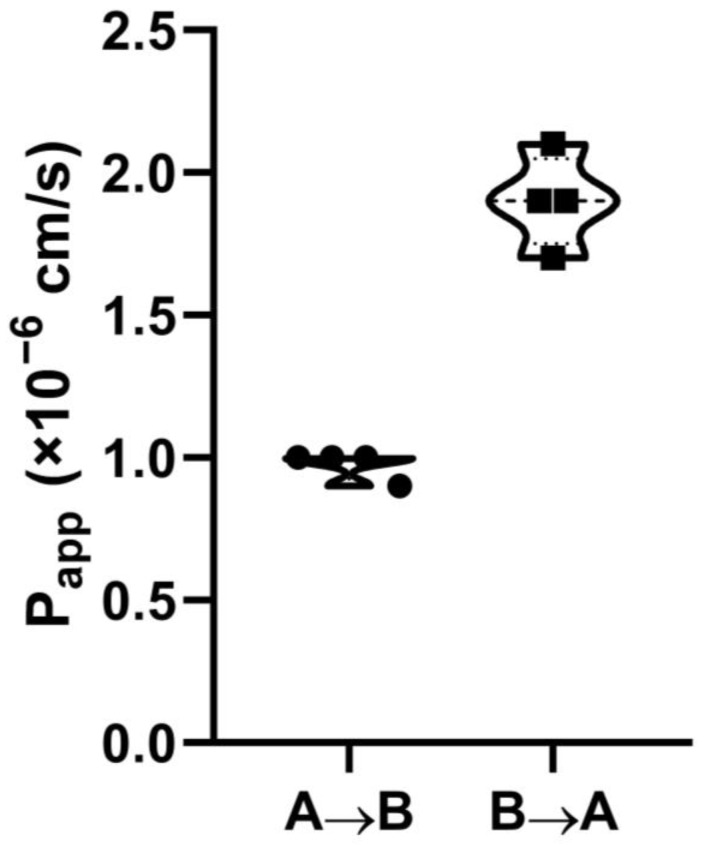
Bidirectional transport of LL-37 through the Caco-2 epithelial monolayer in absorptive (apical-to-basolateral, A → B) and secretory (basolateral-to-apical, B → A) directions.

Human colorectal adenocarcinoma Caco-2 cells are known to be able to form monolayers with a differentiated phenotype, implementing a number of functions proper to the small intestinal villus epithelium. Therefore, polarized monolayers of Caco-2 cells grown on permeable inserts have become the gold standard for in vitro assessment of intestinal drug permeability and absorption [19]. Here, we used the FITC-labeled human cathelicidin LL-37 for an assessment of bidirectional (A → B, absorptive, and B → A, secretory) transport of the host defense peptide through the Caco-2 polarized monolayer. Apparent permeability (P_app_) coefficients in absorptive direction (A → B) for LL-37 were within the range of 0.9–1.0 × 10^−6^ cm/s (Figure 1), which predicted a low absorption of LL-37 in the human gut (0–20%) [20].

A higher P_app_ in the secretory (B → A) direction was observed: 1.7–2.1 × 10^−6^ cm/s for B → A versus 0.9–1.0 × 10^−6^ cm/s for A → B. The uptake (UR) and efflux ratios (ER) were also calculated and shown to be 0.51 and 1.95, respectively. An efflux ratio < 2 is usually considered to indicate the absence of active efflux [21].

### 3.2. The Human Cathelicidin LL-37 Induced Production of IL-8/CXCL8, IL-22, and TNF-β by Caco-2 Cells

In the current study, 48 cytokines, chemokines, and growth factors were assessed in the cell culture media at the protein level by the multiplex xMAP technology (Luminex Corporation, Austin, TX, USA). This technology is based on the simultaneous use of different types of magnetic microspheres. Each type of such microsphere is conjugated with a capture antibody that binds and retains a particular analyte from the sample [22]. Subsequent incubation of the microspheres with detection antibodies and streptavidin–phycoerythrin conjugate is similar to the enzyme-linked immunosorbent assay (ELISA). Thus, the principle of the multiplex xMAP technology is the same as in the ELISA assay except that capture antibodies are conjugated with magnetic microspheres but not coated on polystyrene surfaces. This approach allows the simultaneous evaluation of a wide range of analytes in each sample.

**Figure 2:**
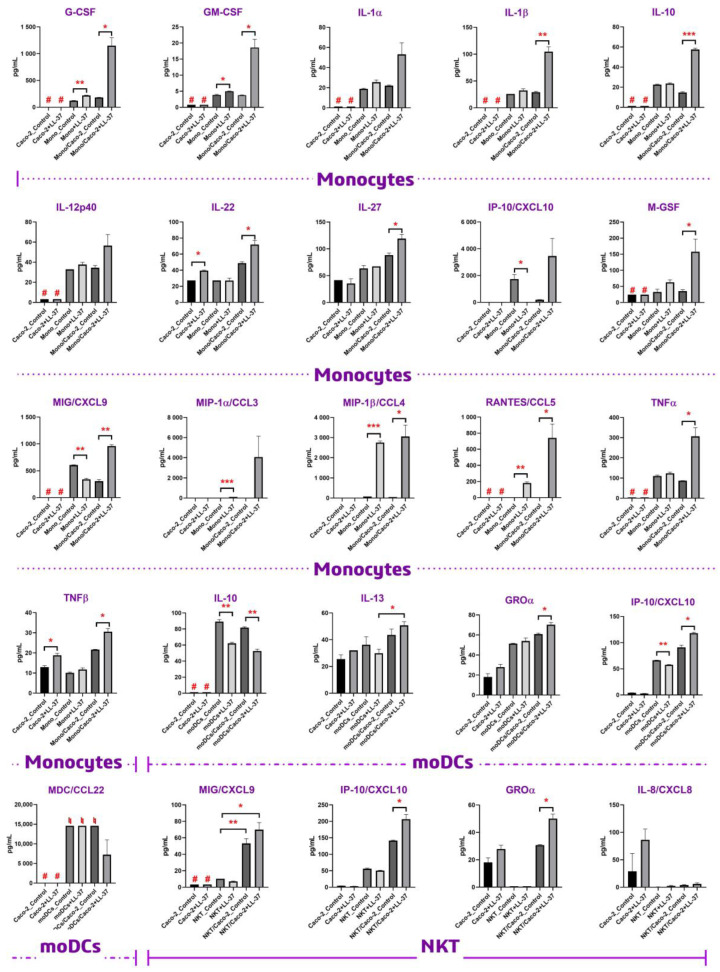
Profiles of cytokines, chemokines, and growth factors levels secreted by human Caco-2 epithelial cells, monocyte-derived dendritic cells (moDCs), NKT cells, and primary blood monocytes in response to stimulation with 1 μM LL-37 (4.5 μg/mL) for 24 h. Error bars represent a standard deviation (±SD) between two replications; # indicates values that fall below the lowest detectable limit; and ♮ indicates values that fall above the highest detectable limit. Significance levels are: * *p* < 0.05, ** *p* < 0.01, *** *p* < 0.0005.

To assess cytokines produced by Caco-2 cells, stimulation with 1 μM LL-37 of the Caco-2 epithelial barrier, grown on a permeable insert with TEER > 400 Ω × cm^2^, was studied. It was shown that LL-37 was able to stimulate the production of IL-8/CXCL8 in Caco-2 cells. Levels of IL-8/CXCL8 were increased from 28 to 86 pg/mL; however, due to a wide range between replications, this elevation failed to reach statistical significance (*p* = 0.16). Nevertheless, it has been previously shown that LL-37 was able to induce the production of IL-8/CXCL8 by epithelial cells, which stimulated neutrophil migration [13]. Interestingly, NKT cells can inhibit the production of IL-8/CXCL8 by epithelial cells (Figure 2). We found that 1μM LL-37 was able to induce a slight but statistically significant elevation of IL-22 and TNF-β levels in the Caco-2 culture media after 24 h of incubation. Thus, LL-37 induced an elevation of IL-22 levels from 27 to 39 pg/mL (*p* = 0.013) in the Caco-2 culture alone. At the same time, LL-37 did not induce any changes in the IL-22 level in the monocyte culture alone (Figure 2). An elevation of IL-22 levels from 48 to 71 pg/mL (*p* = 0.029) in the Caco-2/monocytes co-culture was observed (Figure 2). LL-37 also did not induce any changes in the production of IL-22 by moDCs and NKT cells; however, we observed an elevation of IL-22 levels from 26 to 37 pg/mL (*p* = 0.10) and from 27 to 34 pg/mL (*p* = 0.093) in cases of Caco-2/moDCs and Caco-2/NKT cell co-cultures, respectively (Supporting Materials, Table S1). Similarly, LL-37 induced an elevation of TNF-β levels from 12.9 to 18.9 pg/mL (*p* = 0.02) in the Caco-2 culture alone. No changes in TNF-β levels were observed in the monocyte culture alone, while an elevation of TNF-β levels from 21.6 to 30.5 pg/mL (*p* = 0.015) was observed (Figure 2). No changes in TNF-β production in response to stimulation of moDCs and NKT with LL-37 were observed; however, a slight elevation of TNF-β levels was detected in cases of Caco-2/moDCs and Caco-2/NKT cell co-cultures (Supporting Materials, Table S1).

To the best of our knowledge, we report for the first time that LL-37 can increase the secretion of IL-22 and TNF-β by epithelial cells. It is known that IL-22 is a critical regulator of epithelial homeostasis. It has been shown to be implicated in multiple aspects of epithelial barrier function, including regulation of epithelial cell growth and permeability, production of mucus, antimicrobial peptides (AMPs), and complement [23]. TNF-β, known nowadays as lymphotoxin-α (LTα), has been shown to actively contribute to effector immune responses [24]. LTα regulates dendritic and CD4+ T cell homeostasis in the steady state and determines the functions of these cells during pathogenic challenges [24].

### 3.3. Cathelicidin LL-37 Differently Activates Human Immune Cells in the Absence or Presence of the Caco-2 Epithelial Barrier

Experiments with stimulation of cell cultures were performed in 24-well plates with or without permeable inserts containing the Caco-2 epithelial monolayer. For each immune cell culture, stimulation with 1 μM LL-37 in the presence or absence of the Caco-2 monolayer was studied. Wells containing immune cells alone and ones with the Caco-2/immune cells co-culture, both without LL-37 were used as controls.

It was shown that in some cases, the LL-37 action on immune cells did not depend on the presence of the Caco-2 epithelial monolayer. For instance, stimulation of blood monocytes with LL-37 induced elevation of MIP-1β/CCL4 levels from 78 to 2750 pg/mL (*p* = 0.0004) in the absence of Caco-2 cells and from 49 to above the detectable maximum of 3455 pg/mL (*p* = 0.017) in the presence of Caco-2 cells. Similarly, stimulation of moDCs with LL-37 induced inhibition of IL-10 levels from 89 to 62 pg/mL (*p* = 0.0058) in the absence of Caco-2 cells and from 81 to 52 pg/mL (*p* = 0.0035) in the presence of Caco-2 cells. In some cases, LL-37 induced a slight elevation of the analyte in the absence of Caco-2 cells and a strong elevation in the presence of Caco-2 cells. For example, stimulation of monocytes with LL-37 induced a slight elevation of G-CSF levels from 121 to 220 pg/mL (*p* = 0.002) in the absence of Caco-2 cells and a strong elevation from 181 to 1150 pg/mL (*p* = 0.012) in the presence of Caco-2 cells. Similarly, MIP-1α/CCL3 increased from 45 to 109 pg/mL (*p* = 0.0001) in the absence of Caco-2 cells and from 27 to 3622 pg/mL (*p* = 0.11) in the presence of Caco-2 cells, also in the monocyte culture. In some cases, LL-37 did not induce a statistically significant change in the analyte in the absence of Caco-2 cells but induced its elevation (with the exception of MDC/CCL22 in moDCs, where a decrease in its level was observed) in the presence of Caco-2 cells. For instance, there was a slight statistically insignificant increase in the IL-1β production by monocytes from 25 to 32 pg/mL (*p* = 0.094) in response to incubation with LL-37 in the absence of Caco-2 cells; however, a statistically significant increase in the IL-1β production from 29 to 104 pg/mL (*p* = 0.0072) was observed in the presence of Caco-2 cells (Figure 2). Similar results were obtained for TNFα: elevations of TNFα levels from 110 to 123 pg/mL (*p* = 0.15) for monocytes alone and from 87 to 306 pg/mL (*p* = 0.019) in the presence of Caco-2 cells were observed. The same effects were observed for IL-10: its production increased from 22.5 to 23.6 pg/mL (*p* = 0.23) for monocytes alone and from 14.8 to 57.4 pg/mL (*p* = 0.0007) in the presence of Caco-2 cells. Finally, in some cases, LL-37 induced inhibition of the analyte level in the absence of Caco-2 cells and its elevation in the presence of Caco-2 cells. For instance, stimulation of monocytes with LL-37 induced a decrease in MIG/CXCL9 levels from 606 to 342 pg/mL (*p* = 0.003) in the absence of Caco-2 cells and an increase from 306 to 959 pg/mL (*p* = 0.0018) in the presence of Caco-2 cells. The same was observed for IP-10/CXCL10 produced by monocytes and moDCs (Figure 2).

**Figure 3:**
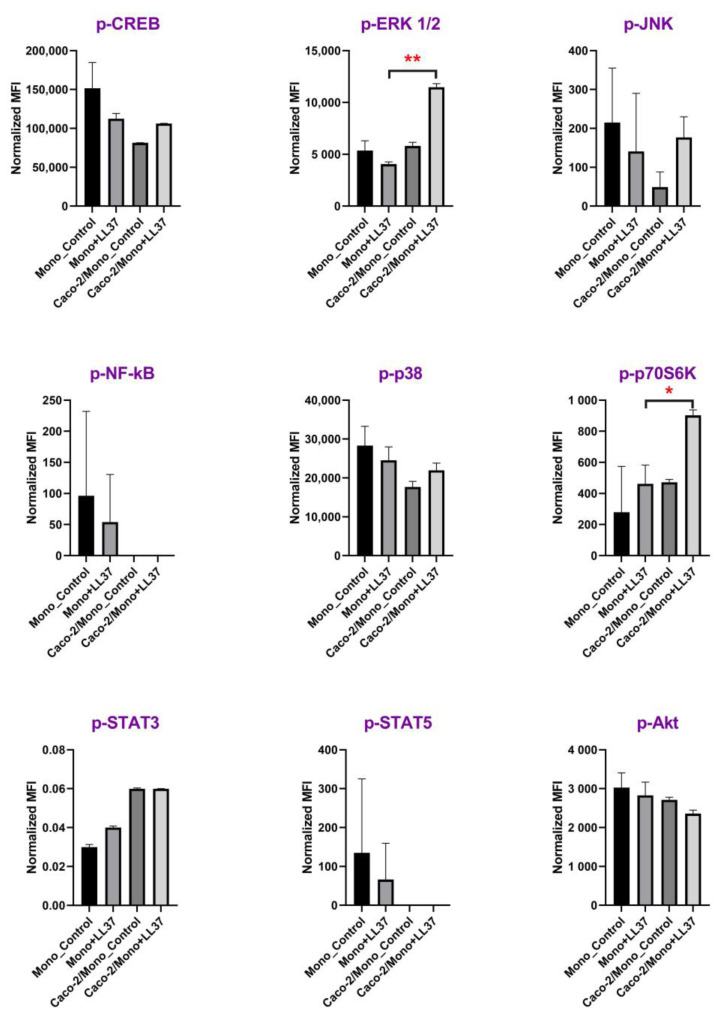
Intracellular signaling in monocytes in the Caco-2/Monocytes co-culture after incubation for 2 h with 1 µM of LL-37 (4.5 μg/mL) as assessed by the multiplex xMAP analysis. Levels of phosphorylated (p-) CREB, ERK 1/2, JNK, NF-κB, p38, p70S6K, STAT3, STAT5, and Akt are expressed as mean fluorescence intensities (MFI) normalized to the β-tubulin level in each sample. Data given as mean normalized MFI ± SD, * *p* = 0.039, ** *p* = 0.0015.

To confirm the role of the epithelial monolayer in the activation of monocytes by stimulation with 1μM LL-37, the phosphorylation of a wide panel of kinases and other intracellular signaling intermediates, including CREB, ERK 1/2, JNK, NF-κB, p38, p70S6K, STAT3, STAT5, and Akt, was studied. It is known that changes in phosphorylation of MAPK can be detected within minutes after exposure to specific stimuli [25]. We chose a 2-hour exposure time to induce MAPK phosphorylation in monocytes and epithelial cells by LL-37 with subsequent production of soluble factors by both cell cultures, which would result in epithelial-dependent activation of monocytes through MAPK phosphorylation. After stimulation for 2 h, an increase in production of phosphorylated ERK 1/2 (Thr185/Tyr187), and p70S6 (Thr412) serine/threonine kinases was detected only in the case of Caco-2/monocytes+LL-37, but not in the cases of monocytes+LL-37 or Caco-2/monocytes (Figure 3; Supporting Materials, Table S2).

## 4. Discussion

The only human cathelicidin, LL-37, has a broad spectrum of biological activities. It is produced at mucosal surfaces by epithelial cells, up-regulated in response to infection and inflammation, and can be released by degranulation of neutrophils. LL-37 has been shown to be involved in human innate and acquired immunity. This host defense peptide displays in vitro antimicrobial activities against many pathogens, including both Gram-positive and Gram-negative bacteria, fungi, protozoans, and enveloped viruses. However, it has been shown to exhibit reduced bactericidal effects under physiological conditions due to the presence of salt and serum. This is why the key role of the direct antimicrobial activity of LL-37 in host defense against microbial infections in vivo is disputable today [26,27]. LL-37 is also involved in tissue repair and exhibits contradictory effects on tumor growth [16]. Moreover, LL-37 has been shown to bind to extracellular DNA and RNA and increase extracellular nucleotide uptake. It can also bind to pathogens and thereby opsonize them, as well as enhance phagocytosis of opsonized pathogens [13]. This peptide has also been shown to attract a variety of leukocytes, such as neutrophils, eosinophils, monocytes, and CD4^+^ T-cells, as well as induce chemotaxis of granulocytes and mast cells.

Despite some limitations, LL-37 is of interest with regard to its clinical applications. For example, phase I clinical trials (NCT02225366) with intra-tumoral injections of LL-37 for melanoma patients with cutaneous metastases have been completed and have shown a significant potency of LL-37 against this form of cancer. A phase IIb double-blind, randomized, placebo-controlled study has shown the effectiveness of LL-37 in the healing of hard-to-heal venous leg ulcers [14]. LL-37 has also been shown to be an effective antiviral drug in the treatment of patients with COVID-19 [15]. Under clinical study, patients with COVID-19 orally took a genetically modified probiotic, *Lactococcus lactis*, which secreted the target ~34 kDa protein, consisting of seven repeats of the mature human LL-37 alternating with enterokinase. Research interest in the therapeutic potential of LL-37 continues to expand, and there might be new findings in the near future [16]. At the same time, there is a lack of data about LL-37 absorption in the gut in vivo or its permeability through the gastrointestinal barrier in vitro. This is why the first step of the current study was to evaluate the ability of LL-37 to transfer across the Caco-2 monolayer. We demonstrated that LL-37 can permeate through the Caco-2 monolayer with a low efficiency (P_app_ = 0.9–1.0 × 10^−6^ cm/s), which may predict 0–20% absorption in the gut [20]. Our data indicate that drug delivery systems, for example, absorption enhancers, might be required for the intact LL-37 to increase its bioavailability in the case of oral administration.

LL-37 is considered today as a potential drug with pronounced immunomodulatory activity. There are plenty of studies on LL-37’s immunomodulatory activities in vitro on different types of cells [28]. However, it still remains an open question whether immunomodulatory activities observed in vitro take place in vivo, or, in other words, whether the immunomodulatory effects of LL-37 are physiologically relevant. A major criticism of host defense peptide research is that antimicrobial and immunomodulatory effects are observed in cell cultures at much higher concentrations than those expected to occur in vivo or observed only at sites of severe chronic inflammation [27]. It has been demonstrated that the average concentration of the human cathelicidin LL-37 was 1.18 µg/mL in the serum of healthy individuals, which was several folds higher than that for other neutrophil-specific granule proteins [29]. However, local concentrations of LL-37 upon neutrophil degranulation can be much higher [27]. LL-37 has been detected at concentrations of ∼5 μg/mL in the bronchoalveolar lavage fluid of healthy infants, and its concentration was increased by two- to threefold in the bronchoalveolar lavage fluid of infants with systemic or pulmonary inflammation [30]. In this regard, the chemotactic ability of LL-37 is not impaired by serum, and LL-37 is able to attract monocytes and mast cells and induce migration of epithelial cells at the low physiological concentration of 5 µg/mL [13]. At the same time, activation of granulocytes and stimulation of mast cell degranulation require a concentration of 10 µg/mL, while induction of neutrophil extracellular trap (NET) formation takes place at a concentration of 25 µg/mL [31,32], which demands elevated levels of LL-37.

In contrast to the chemotactic potential of LL-37, available data on its ability to induce cytokine production by different cells argue for the absence of its biological activity at low physiological concentrations of ∼5 μg/mL observed in non-inflamed tissues. Previously, it has been reported that LL-37 was not able to increase the production of TNFα or IL-10 in human PBMCs at a concentration of 5 μg/mL [33]. In the current study, we used LL-37 at a concentration of 1 μM (4.5 μg/mL) to study whether this peptide had immunomodulatory activity under non-inflamed physiological conditions. In our study, we did not observe upregulation of either TNFα or IL-10 upon direct stimulation of monocytes with 4.5 μg/mL LL-37, which is in accordance with previous data. However, we showed that LL-37 at this concentration was able to induce the production of both TNFα and IL-10 by monocytes in the presence of the Caco-2 epithelial barrier (Figure 2). Similar results have been obtained in the case of the main monocytic pro-inflammatory cytokine, IL-1β. Previous data denoted that LL-37 could stimulate the production of IL-1β by monocytes only at concentrations of 10–20 μg/mL. These values fall within the LL-37 concentration range measured during inflammation [34], but not under non-inflamed physiological conditions. Our data neither showed significant upregulation of IL-1β upon direct stimulation of monocytes with 4.5 μg/mL LL-37. However, 4.5 μg/mL LL-37 was shown to be sufficient for activation of primary monocytes and secretion of IL-1β by them in the presence of the Caco-2 monolayer. This fact allowed us to assume that epithelial-immune cell crosstalk might have a predominant role in the biological action of LL-37 in vivo, making this peptide able to activate immune cells at lower concentrations while being close to epithelial barriers. This phenomenon was observed not only in the monocytic culture but also in other cultures of immune cells, such as NKT and dendritic cells. However, this effect on the latter cultures was less pronounced (Figure 2). To confirm our assumption, we decided to obtain one more piece of biological evidence at the cell signaling pathway level. Activation of signaling pathways only in the case of Caco-2/monocytes+LL-37, but not in the cases of monocytes+LL-37 or Caco-2/monocytes, may argue for epithelial-immune cells crosstalk-mediated cell activation by LL-37. For this experiment, the culture of primary monocytes was chosen, and a broad panel of kinases and other signaling intermediates, including CREB, ERK 1/2, JNK, NF-κB, p38, p70S6K, STAT3, STAT5, and Akt, was used. Consequently, 2 h after stimulation with 4.5 μg/mL of LL-37, we found in cell lysates a significant phosphorylation of extracellular signal-regulated kinase 1/2 (ERK1/2) and p70S6 kinase only in the case of Caco-2/monocytes+LL-37, but not in the cases of monocytes+LL-37 or Caco-2/monocytes (Figure 3; Supporting Materials, Table S2). Thus, the MAPK/ERK pathway (also known as the Ras-Raf-MEK-ERK pathway) was activated in monocytes by 4.5 μg/mL LL-37 due to communication between epithelial cells and monocytes. Then, ERK1/2 kinase probably phosphorylates p70S6 kinase, whose substrate is the S6 ribosomal protein. Phosphorylation of S6 induces protein synthesis by ribosomes and is a rate-limiting step for translation. Previously, it has been shown that LL-37 could signal via the induction of phosphorylation of the mitogen-activated protein kinases (MAPK) ERK1/2 and p38 in peripheral blood-derived monocytes and in the human bronchial epithelial (HBE) cell line [35]. It is important to note that the activation of ERK1/2 and p38 was observed only at high concentrations of LL-37 (25–50 μg/mL). However, in the presence of GM-CSF, activation of these kinases was shown to occur at lower concentrations of LL-37 (5–10 μg/mL) [35]. Thus, in the presence of a secondary signal such as GM-CSF (but not G-CSF), the threshold of the LL-37 concentration for displaying its immunomodulatory action may decrease. Our results (Figure 2, Supporting Table S1) and data obtained by Urdinguio et al. [36] indicated that the Caco-2 cell line is not a source of GM-CSF. We assumed that LL-37 induced the production of some molecular factors in the Caco-2/monocyte co-culture that acted as GM-CSF, decreasing the LL-37 concentration threshold required for the activation of monocytes. Interestingly, we did not observe phosphorylation of p38 in monocytes stimulated for 2 h in the absence or presence of LL-37 (Figure 3). In contrast to the p70S6 kinase, which can be a part of the MAPK/ERK pathway, p38 is a part of the stress-activated protein kinase (SAPK) pathway. The MAPK/ERK pathway is classically activated by growth factors and mitogens, while the p38-MAPK/SAPK pathway can also be activated by stress factors and inflammatory cytokines [37]. Apparently, LL-37 is able to activate both MAPK/ERK and p38-MAPK/SAPK pathways at high concentrations (25–50 μg/mL), while at low physiological concentrations (4.5 μg/mL), LL-37 can induce epithelial-dependent activation of the only MAPK/ERK pathway in blood monocytes.

## 5. Conclusions

In summary, we assessed the permeability of the human cathelicidin LL-37 across the Caco-2 polarized monolayer, which is a model of the intestinal epithelial barrier. Here, we showed that this peptide can permeate through the Caco-2 monolayer with a low efficiency (P_app_ = 0.9–1.0 × 10^−6^ cm/s), which predicts its low (0–20%) absorption in the gut. By using the Caco-2/immune cell co-culture, we demonstrated the crucial role of epithelial-immune cell crosstalk in exhibiting the immunomodulatory properties of the human cathelicidin LL-37, which might shed light on its biological action in vivo. For the first time, our data show that LL-37 can increase the secretion of IL-22 and TNF-β by epithelial cells. We also demonstrated that LL-37 at a low physiological concentration of 4.5 μg/mL is able to induce a strong production of only MIP-1β/CCL4, but cannot fully activate monocytes. However, at the same concentration, LL-37 can fully activate monocytes in the Caco-2/immune cells co-culture model, inducing intracellular phosphorylation of ERK1/2 and p70S6 serine/threonine kinases, as well as the production of cytokines IL-1, IL-10, IL-27, TNFα, chemokines MIP-1, MIG, IP-10, RANTES, and growth factors G-CSF, GM-CSF, and M-CSF. Our study suggests the existence of some molecular factors produced by stimulated epithelial cells that probably activate the same MAPK/ERK signaling pathway in monocytes, thus decreasing the LL-37 concentration threshold for the activation of monocytes. We also demonstrated similar effects at the cytokine production level on other cell cultures, such as monocyte-derived dendritic (moDCs) and NKT cells; however, in other cell cultures this effect was less pronounced. These findings indicate that the human cathelicidin LL-37 is able to fulfill its immunomodulatory action in vivo at low physiological concentrations of <5 μg/mL in the presence of epithelial barriers, at least near the intestinal epithelium.

## Data Availability

All data generated and analyzed during this study are included in this published article and its Supplementary Materials file.

## Supplementary Materials

The following supporting information can be downloaded at: https://www.mdpi.com/article/10.3390/biom13091316/s1, Figure S1: Isolation of NKT cells (CD3+CD56+) by cell sorting; Table S1: Absolute levels of 48 cytokines, chemokines, and growth factors assessed by multiplex xMAP technology; Table S2: Qualitative assessment of phosphorylated analytes in monocytic cell lysates by multiplex xMAP technology.
