# Supplementary figures and images for: Epithelial-Immune Cell Crosstalk Determines the Activation of Immune Cells In Vitro by the Human Cathelicidin LL-37 at Low Physiological Concentrations
Source: Biomolecules. 2023 Aug 28;13(9):1316. doi: 10.3390/biom13091316 (PMC10526274; doi:10.3390/biom13091316)

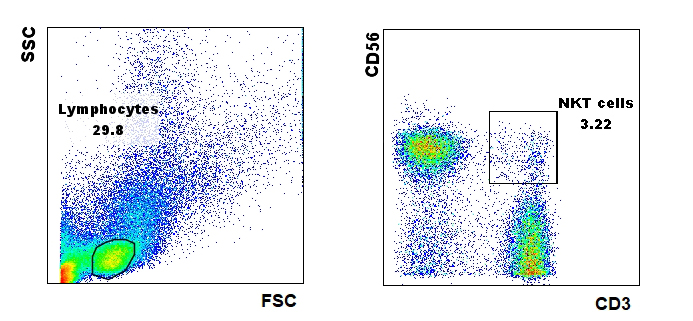

Supplement: Supplementary file 1 [file biomolecules-13-01316-s001.zip › Figure S1.jpg]
